# Photovoltaic-driven organic electrosynthesis and efforts toward more sustainable oxidation reactions

**DOI:** 10.3762/bjoc.11.32

**Published:** 2015-02-23

**Authors:** Bichlien H Nguyen, Robert J Perkins, Jake A Smith, Kevin D Moeller

**Affiliations:** 1 Washington University in Saint Louis, Saint Louis, Missouri 63130, United States

**Keywords:** electrochemistry, sustainable oxidation reactions, visible light

## Abstract

The combination of visible light, photovoltaics, and electrochemistry provides a convenient, inexpensive platform for conducting a wide variety of sustainable oxidation reactions. The approach presented in this article is compatible with both direct and indirect oxidation reactions, avoids the need for a stoichiometric oxidant, and leads to hydrogen gas as the only byproduct from the corresponding reduction reaction.

## Introduction

Organic electrochemistry is an extremely versatile tool for conducting a wide variety of chemical reactions [1–3]. This versatility stems from both the gentle, acid/base neutral reaction conditions employed for the reactions and the adjustable potential of the working electrode that enables the oxidation and reduction of substrates that often greatly differ in their electronic structure.

It is particularly easy to take advantage of the opportunities electrochemistry offers when conducting constant current (galvanostatic) electrolysis [4]. When a constant current is passed through an electrolysis cell, the potential at the anode increases until it reaches that of the substrate in solution with the lowest oxidation potential. It then remains constant at that potential until the effective concentration of the substrate at the anode decreases to the point that the rate of substrate oxidation is small relative to the rate of electron transfer. At that point, the potential at the anode begins to increase and the selectivity of the reaction for the initial substrate is lost. When a low current density is used for the reaction, over 90% of the initial substrate can be consumed before this loss of selectivity occurs. Hence, at low current densities a constant current electrolysis reaction automatically adjusts to the potential of the substrate to be oxidized and then remains at that potential for the majority of the reaction. In this way, a series of substrates can be selectively oxidized using the same reaction conditions even if the substrates have significantly different oxidation potentials. For most of the following cases discussed, reticulated vitreous carbon (RVC) is used as a highly porous anode material to keep current densities low. All of the RVC electrodes used were of 100 pores per inch and approximately 1 × 1 cm in size, and thus the differences in current are directly proportional to the current density from experiment to experiment. An equal but opposite reduction reaction happens at the cathode. For all of the oxidation reactions highlighted in this work, this reduction reaction leads to the formation of hydrogen gas.

**Scheme 1:**
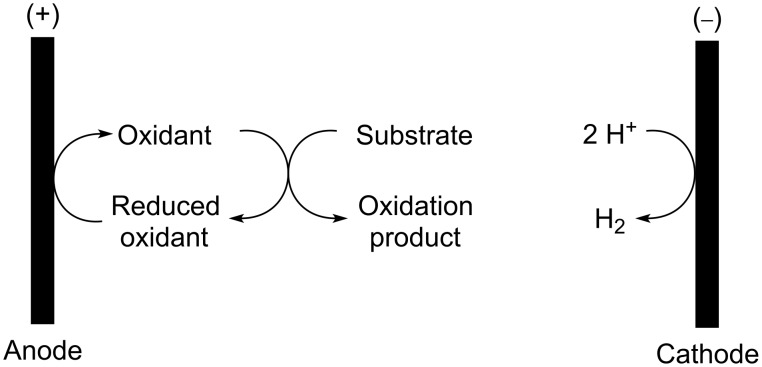
Electrochemical recycling of a chemical oxidant.

In addition to the direct oxidation of a substrate described in the preceding paragraph, indirect electrochemical methods also offer a powerful means of recycling chemical oxidants [5]. In such experiments, the potential at the anode increases to a point where it matches the oxidation potential of the reduced chemical oxidant (Scheme 1). The reduced chemical oxidant is then oxidized in order to generate the active chemical oxidant. The chemical oxidant then performs the desired chemical transformation before returning to the anode as its reduced form. The process converts the chemical oxidant into a catalyst. Since the oxidant is not consumed during the reaction, the potential at the anode remains constant throughout the electrolysis. As in the direct oxidation, the corresponding reduction reaction at the cathode generates hydrogen gas. Hence, the reactions allow for the use of a chemical oxidant together with its inherent selectivity while avoiding the byproducts associated with consumption of the reagent.

Based on this scenario, it is tempting to suggest electrochemistry is a “green method”. However, any attempt to promote electrochemistry as being environmentally benign must account for both the use of the electrolyte in the reactions and the source of the electricity used.

Most electrochemical reactions require the use of an electrolyte. The electrolyte provides counter ions for the ions generated at the electrodes and serves to reduce the resistance of the cell by making the electron-transfer reaction at the electrodes easier. The presence of this electrolyte, often used in large excess, can render an electrochemical reaction less than sustainable unless the electrolyte is recycled. A number of research groups have addressed this issue by either providing alternative electrolytes that can be easily recycled [6] or conducting the reactions in ionic liquids [7]. An alternative approach takes advantage of flow technology. In these experiments, the electrolysis reaction solution is flowed as a thin film between two closely spaced electrodes. The small separation between the electrodes enables the charged molecules generated at each electrode to interact. This neutralizes the charges, reduces the resistance of the cell, and eliminates the need for an electrolyte [8].

**Figure 1:**
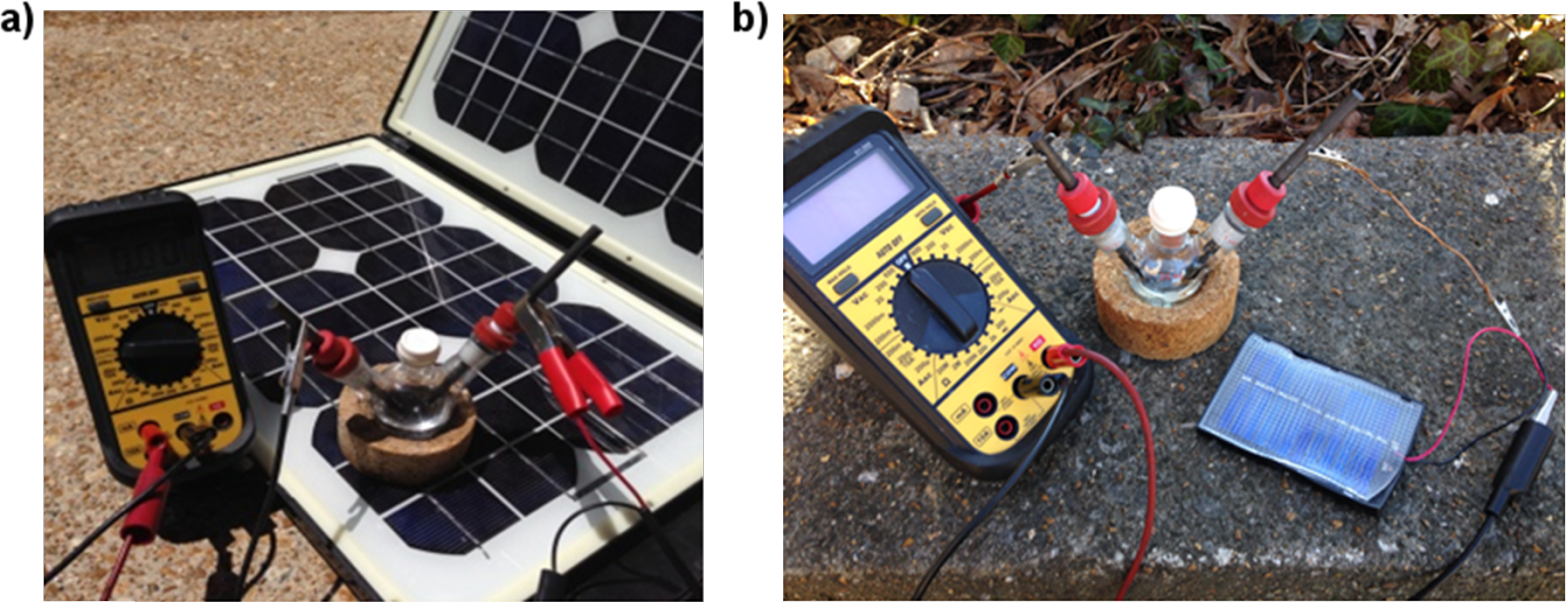
a) Electrolysis setup with a “suitcase” photovoltaic device. b) Electrolysis with a very simple, commercially available, photovoltaic cell.

With efforts to address the electrolyte problem already underway in the community, we turned our attention to the source of electricity. Since the potential at the electrodes in a constant current electrolysis automatically adjusts to match that of the substrates, in principle, any source of current can be used to drive the reactions in a selective fashion. With this in mind, it seemed that a photovoltaic system would make an excellent power supply for performing the reactions with minimal environmental impact. There are numerous commercial photovoltaic systems that can be used to convert visible light into electricity, two of which are shown in Figure 1 [9].

The first is a portable photovoltaic device capable of generating a potential large enough to recharge a wide variety of batteries. The second is a much less expensive alternative that is sold for use in connection with science fair projects and the operation of solar-driven toys [10]. Both can be used to power an electrochemical reaction, and both offer the opportunity to perform oxidation reactions that consume only sunlight and generate hydrogen gas as the only byproduct.

The experimental setup for a photochemically driven reaction is trivial. One simply needs to connect the wires originating from the photovoltaic source to the electrodes used for the reaction. One can vary the current passing through the cell by simply changing the amount of photovoltaic that is exposed to light. With a large panel like the one shown in Figure 1a, regions of the array can be covered to generate only a small amount of current. For the smaller units (Figure 1b), the total surface area of photovoltaic can be controlled by varying the number of individual photovoltaic cells connected in series. One is, thus, not limited by the day to day variations in sunlight intensity, as the current through the cell can be adjusted very quickly using these methods.

With an experimental design in place, a series of direct and indirect oxidation reactions were used to determine the viability of the method [11–12].

## Discussion

### Direct oxidation

**Scheme 2:**
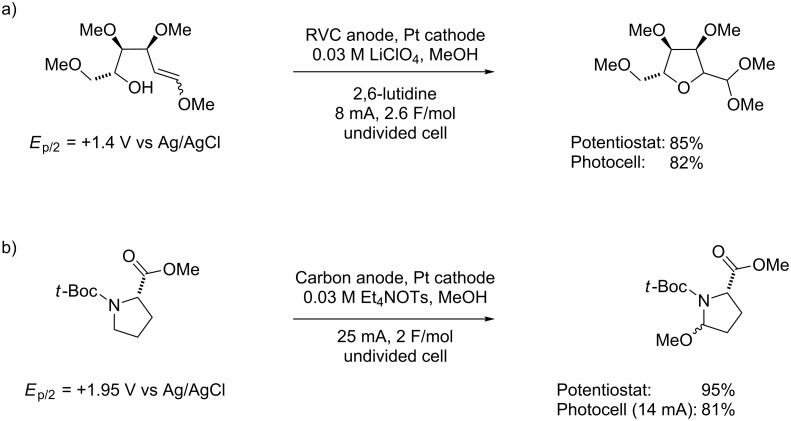
Examples of solar-driven direct electrochemical oxidations.

Initial efforts began by examining reactions where the substrate to be oxidized underwent the electron-transfer reaction directly at the electrode surface. We have employed reactions of this nature to functionalize amides [13–14] and to conduct umpolung reactions [15–16] that originate from electron-rich olefins [17]. Two examples are given in Scheme 2 [11]. A yield for the reactions is given for an experiment using photovoltaics as the power supply and a comparable reaction using a more traditional electrochemical setup. The photovoltaic-based reactions were conducted by adjusting the area of the photovoltaic cell exposed to the light until the current passing through the reaction was the same as that used with the traditional setup. The two cases in Scheme 2 were selected because the oxidation potential of the substrates differed by more than 0.5 V. However, that difference in potential had little effect on the success of the electrolysis reactions, as the potential at the anode surface adjusted to that of the substrate. In both cases, the reaction using the photovoltaic system led to excellent product yield. In the case of the amide oxidation, a lower current was passed through the cell for the photovoltaic relative to the traditional setup. The lower current was a result of limitations associated with the very simple photovoltaic system employed (Figure 1b). The inexpensive photovoltaic setup did not produce a large enough potential drop to overcome the resistance of an electrolysis reaction with a more difficult-to-oxidize substrate. The use of a larger photovoltaic cell would have afforded a larger potential drop across the cell, a scenario that would allow for the passage of more current through the cell. This approach would have been undertaken if further optimization of the reaction had been needed. Another point of interest is related to the choice of the electrolyte for the reaction. The reactions highlighted in Scheme 2 provide an opportunity to address this issue. Typically, the choice of electrolyte is not crucial, and tetraethylammonium tosylate can be employed for the majority of reactions reported. However, this is not always the case. In the first reaction shown, LiClO_4_ was used as the electrolyte when a reaction utilizing Et_4_NOTs failed to afford the product [18]. The change was made because of the polarity of the substrate. In an electrolysis reaction, the electrolyte forms a double layer immediately around the electrode surface that can prevent molecules from reaching the electrode. For example, a “greasy” hydrocarbon-based electrolyte will form a hydrophobic double layer and exclude polar molecules from the region surrounding the electrode. This was the case when Et_4_NOTs was used as the electrolyte for the oxidation of the sugar derivative (Scheme 2a). The result was a dramatic reduction in the current efficiency of the process. The switch to LiClO_4_ as the electrolyte led to a more hydrophilic double layer that no longer excluded the sugar-based substrate, leading to an improved current efficiency and a high product yield.

**Scheme 3:**
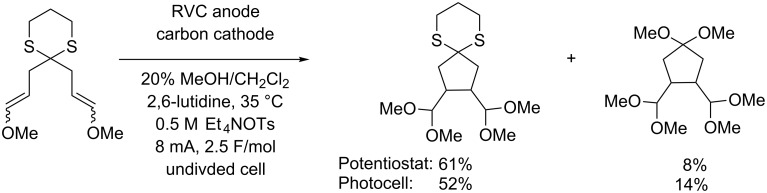
Overoxidation of dithioketal.

A number of direct electrolysis reactions were driven by visible light using the same approach shown in Scheme 2. In almost every case, the simple visible-light-driven electrolysis setup appropriately mimicked reactions performed with the significantly more sophisticated electrochemical equipment. The examples where the simple electrolysis setup was not as effective typically required more careful control of the current and hence the working potential of the electrode. The reaction in Scheme 3 provides an example of such a reaction. In this reaction, the initially formed cyclic product has an oxidation potential that is not significantly higher than that of the starting material. As a result, undesired oxidation of the product and cleavage of the dithioketal moiety were observed. When a photovoltaic device was used to conduct the reaction, an increase in this over-oxidation product was observed, presumably due to lessened control of the current being passed through the reaction.

### Indirect oxidation

**Scheme 4:**
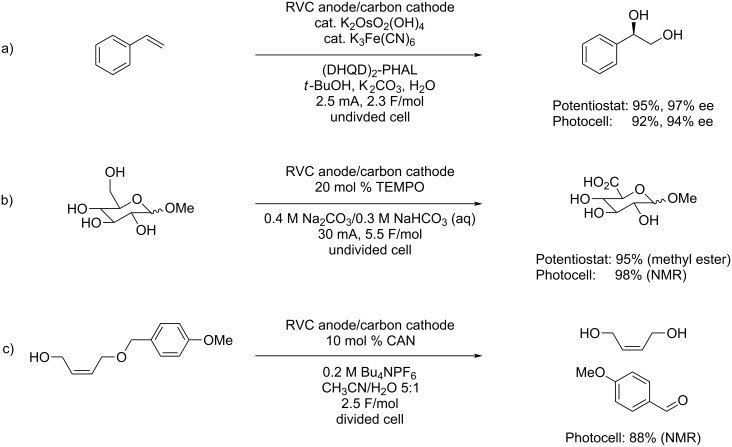
Examples of solar-driven, indirect electrochemical oxidations.

While direct oxidation reactions can be powerful synthetic tools, electrochemical reactions are typically more selective and are based on the relative oxidation potential of the various groups in solution. The group with the lowest oxidation potential is the group that will be oxidized. Chemical oxidations, however, do not have this limitation. They can be selective for one substrate based on steric effects, chirality, or other factors. For this reason, the sunlight-driven oxidation reactions were extended to the recycling of chemical oxidants. Three examples are shown in Scheme 4 [12] where each was chosen for its unique feature related to the indirect electrochemical approach.

In the first reaction (Scheme 4a), an asymmetric dihydroxylation with K_2_OsO_2_(OH)_4_ and hydroquinidine 1,4-phthalazinediyl diether ((DHQD)_2_PHAL) was performed in a catalytic fashion by recycling the ferricyanide cooxidant at the anode [19]. In this case, the use of the chemical oxidation strategy allows for incorporation of an asymmetry-inducing element into the transition state for the oxidation in a manner not possible with a direct electrochemical oxidation.

The oxidation proceeded smoothly in the light-driven electrochemical reaction. The yield and ee of the product was in accordance with that reported in the literature for the reaction using a traditional electrochemical setup. The same electrochemical solar cell developed for the direct oxidation experiments could be utilized to conduct indirect electrolysis.

In the second oxidation illustrated, 2,2,6,6-tetramethylpiperidin-1-oxyl (TEMPO) was recycled at the anode [20]. The bulky oxidant was used to selectively oxidize the primary alcohol of a glucose derivative in the presence of the unprotected secondary alcohols. Once again, this is an example of selectivity that cannot be accomplished with a direct electrochemical oxidation, which would select the oxidation based exclusively on potential. Again, the yield from the light-driven process is comparable to the literature value for the oxidation.

The final oxidation in Scheme 4c illustrates the use of the light-driven electrolysis reaction for recycling ceric ammonium nitrate (CAN) for a *p*-methoxybenzyl deprotection of an alcohol proceeding through oxidation of the aromatic ring. This is a reaction that does not have a direct literature precedent on a preparative scale. Instead, it is an electrochemical reaction that was initially developed in the context of performing site-selective reactions on a microelectrode array [21]. It was selected for discussion here because the reaction serves to both illustrate the range of oxidation reactions that can be performed in a catalytic fashion using a simple photovoltaic and highlight the scalability of the process. In order to perform the reaction on a preparative scale, the array-based method was increased by twelve orders of magnitude without any change in the overall reaction conditions.

### Recent advances

The reactions illustrated above are an ideal set. Each was selected to address a key scientific point and because the electrochemical reaction used was straightforward and not particularly complex. The reactions were all easily performed at room temperature and were conducted in simple electrochemical cells and governed by the initial electron-transfer reaction. Given that not all electrochemical oxidation reactions are so straightforward, the compatibility of such a simple photovoltaic power supply with a more challenging electrolysis reaction is a valid concern. In the following section, the use of the visible-light-driven electrolysis setup for three such reactions is illustrated.

**Scheme 5:**
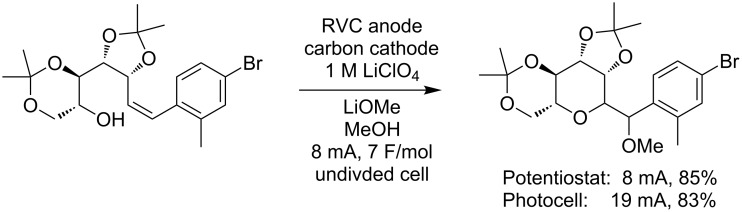
Solar-driven synthesis of *C*-glycosides.

The first case stems from the use of an anodic coupling reaction to make *C*-glycoside derivatives from styrenes (Scheme 5) [22]. We have found that the yield of product obtained in these reactions is directly dependent on the efficient removal of a second electron from the system. This requires higher current densities at the electrodes and often the use of more electrolyte. Neither requirement is a problem for the visible-light-driven electrolysis system. The reaction using the photovoltaic power supply provided the same result as the electrolysis using a more advanced electrochemical setup.

**Scheme 6:**
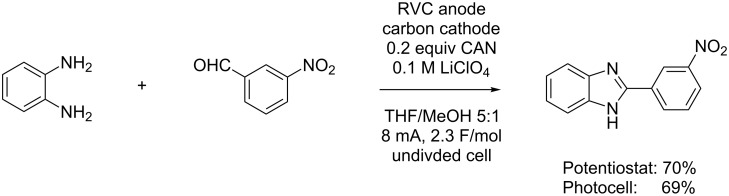
Solar-driven oxidative condensation.

The second example (Scheme 6) involves an oxidative condensation reaction between an aromatic aldehyde and a diamine [23–25]. The reaction requires a careful balance between the initial condensation reaction and the oxidative step with either CAN or DDQ serving as the mediator.

**Scheme 7:**
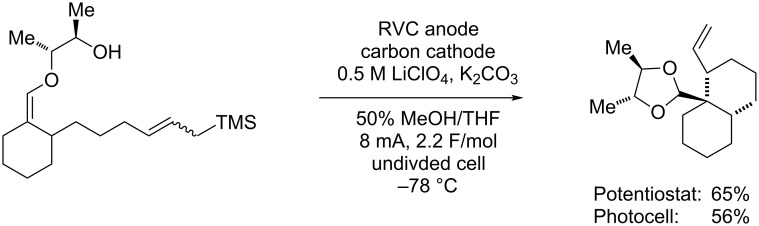
Solar-driven oxidative cyclization with a second nucleophile.

In the third reaction (Scheme 7), an intramolecular alcohol nucleophile was added to an olefin coupling reaction [26]. When a radical cation was generated from the enol ether, it was rapidly trapped by the alcohol nucleophile. This generated a radical that was in turn trapped by an allylsilane. The loss of a second electron and elimination of the silyl group led to the final product. To be successful, the reaction needed to overcome the barriers of quaternary carbon and six-membered ring formation. The use of the second nucleophile and a fast initial trapping reaction reduced the cation character of the radical cation intermediate, slowed competitive elimination reactions, and allowed for the desired quaternary carbon formation. In these reactions, the initial alcohol trapping reaction was found to be both exothermic and reversible. Hence, cooling the reaction to −78 °C helped to maintain the initial cyclization and to keep the cation character of the reactive intermediate low. This significantly increased the yield of the desired cyclization.

As in the previous cases, none of these complications (or the need for the lower reaction temperature) prevented the use of the very simple reaction setup. The use of the photovoltaic system with visible light to generate the electricity needed for the reaction led to product yields only slightly less than those obtained when the overall system was more carefully controlled.

## Conclusion

A broad range of electrochemical oxidations can be performed in a fashion that consumes only visible light and generates hydrogen gas as the only byproduct. The reactions include both direct and indirect oxidation strategies, which can be used to generate new carbon–carbon bonds, functionalize amides, and capitalize on the reagent-based selectivity associated with chemical oxidants. In all cases, the use of constant current electrolysis conditions allows the potential at the anode surface to be adjusted to that of the substrate. Hence, the same experimental protocol can be used for each reaction.

It should be noted, that in many ways, the use of a simple photovoltaic device to directly power an electrochemical reaction as illustrated in Figure 1 is a gimmick. For a more complex system or large-scale electrolysis, a more sophisticated photovoltaic array would be used to harvest enough energy to run a standard potentiostat. This would result in a far more selective electrolysis since the current passed through the reaction could be carefully controlled and the efficiency of the electrochemical process optimized. However, the use of a simple photovoltaic device to drive the reactions does highlight two key points. First, the reactions illustrate how electrochemistry can be used to expand the growing area of visible-light-driven chemistry to include electron-transfer reactions in molecules and reaction systems that have no internal chromophore. Second, the reactions illustrate how simple sustainable electrochemical methods can be employed. This is particularly important since the larger synthetic community is often hesitant to adopt electrochemical reactions. This hesitation frequently has its origins in the perception that electrochemical reactions require the use of sophisticated and expensive equipment. The range of reactions that can be conducted with the very simple reaction setup shown in Figure 1b demonstrates that this perception is not accurate. Any electrochemical reaction in the literature can be mimicked satisfactorily with only a small investment of time and money.

## Experimental

### General information

Electrolysis reactions were performed using a photovoltaic cell and a light source (direct sunlight or a compact fluorescent bulb (hydrophonic, full spectrum, 60 W, 5500K)) with an ammeter and an optional coulometer connected in series. The output voltage of the photovoltaic cells varied from 6–35 V depending on the light intensity, which was varied to control the current output. For reactions requiring higher current, a Topray solar panel briefcase was connected in series with the reaction flask (Figure 1a). Alternatively, several 6 V solar photovoltaic cells can be connected in series to generate the equivalent amount of current (Figure 1b).

### Representative procedure for solar-driven direct electrochemical reactions (Scheme 2a)

The enol ether substrate was dissolved in anhydrous MeOH (0.03 M) with lithium perchlorate (0.03 M, 1.0 equiv) in a flame-dried three-necked round-bottomed flask at room temperature under an argon atmosphere. The flask was equipped with a RVC anode and platinum wire cathode using two of the three necks of the flask. The photovoltaic system was inserted in series with the reaction flask along with an ammeter to monitor the current. The reaction was carried out at a constant current of 8.0 mA until the desired amount of charge was passed. The crude mixture was washed with water and then the organic solution was concentrated under reduced pressure and purified by silica gel chromatography.

### Representative procedure for solar-driven indirect electrochemical reactions (Scheme 6)

A flame-dried three-necked round-bottomed flask was charged under argon with 1 equiv of *o*-phenylenediamine (0.31 mmol, 33.8 mg), 1.1 equiv of 3-nitrobenzaldehyde (0.34 mmol, 52 mg), 20 mol % CAN (31 mg), and LiClO_4_ (0.1 M, 127 mg) in 12 mL of THF/MeOH 5:1. A RVC anode and a carbon rod cathode were inserted into the flask. A constant current of 8 mA was supplied (either via a potentiostat or a photovoltaic cell) until 2.3 F/mol of charge had passed. After the electrolysis, the contents of the flask were extracted with EtOAc, washed with brine, and dried with MgSO_4_. The product was purified by column chromatography (EtOAc/hexanes 1:1) to give the benzimidazole product.

Specific electrolysis procedures for other substrates may be found in the original publications for the reactions as cited in the main text. The modification of these reactions to the solar-driven versions were carried out according to the general information and example solar-driven procedures provided above.
